# Rechtliche, fachliche und strukturelle Aspekte einer modernen akutstationären Versorgung von Kindern und Jugendlichen mit psychischen Störungen

**DOI:** 10.1007/s00103-024-03852-2

**Published:** 2024-03-08

**Authors:** Timo D. Vloet, Julia Geißler, Regina Taurines, Thomas Jans, Arne Bürger, Marcel Romanos

**Affiliations:** Zentrum für Psychische Gesundheit (ZEP), Klinik und Poliklinik für Kinder- und Jugendpsychiatrie, Psychosomatik und Psychotherapie, Universitätsklinikum Würzburg, Margarete-Höppel-Platz, 97084 Würzburg, Deutschland

**Keywords:** Kinder- und Jugendpsychiatrie, Geschlossene Unterbringung, § 1631b BGB, Akutversorgung, Krisenmanagement, Child and adolescent psychiatry, Protective intensive care, Mental health care, § 1631b BGB, Crisis management

## Abstract

In den letzten Jahren ist der Anteil der Notfallvorstellungen in den Versorgungsstrukturen für Kinder und Jugendliche kontinuierlich gestiegen. Die akutstationäre geschlossene Behandlung ist ein wesentlicher Baustein der Versorgung und dient vorrangig dem unmittelbaren Schutz von Kindern und Jugendlichen in psychischen Krisensituationen. Die geschlossene Unterbringung unterliegt strengen rechtlichen Voraussetzungen, die 2017 in Form des § 1631b BGB novelliert wurden und seither erstmals zu einer klaren Trennung der Anwendung von freiheitsentziehenden Maßnahmen (FEM) gegenüber der freiheitsentziehenden Unterbringung (FEU) von Kindern und Jugendlichen geführt hat.

Anhand der Umgestaltung der Intensiveinheit am Universitätsklinikum Würzburg werden in diesem Artikel beispielhaft die Erfordernisse an eine moderne Akutversorgung von Kindern und Jugendlichen mit psychischen Erkrankungen dargestellt. Infolge der Modernisierung am Universitätsklinikum Würzburg konnte Freiheitsentzug auf ein Minimum reduziert werden – auf aktuell durchschnittlich 1,5 Tage bei etwa 500 Aufnahmen mit FEU im Jahr. Die Gefahr der Hospitalisierung insbesondere bei chronisch suizidalen Patienten besteht praktisch nicht mehr. Durch Kooperation mit anderen Kliniken ist es in der Region seit 2017 gelungen, alle minderjährigen Patienten mit akutstationärem Behandlungsbedarf in kinder- und jugendpsychiatrischen Strukturen zu versorgen. Langfristige geschlossen-stationäre Behandlungen über viele Monate kommen bei chronischer Suizidalität nicht mehr vor.

## Abstract (en)

Recent years have seen a continuous rise in the proportion of emergency contacts across all mental health-related care structures for children and adolescents. Treatment in a protective intensive care unit constitutes an essential element of care and primarily serves the immediate protection of children and adolescents during mental health crises. Protective intensive care is subject to strict legal requirements. Those requirements were amended in 2017 via changes to § 1631b BGB (German Civil Code), leading to a clear separation of the stay in protective intensive care per se and the use of coercive measures. Using the restructuring of the intensive care unit of the Clinic for Child and Adolescent Psychiatry, Psychosomatics and Psychotherapy at the University Hospital Würzburg as an example, the article illustrates the requirements for modern acute care of children and adolescents with mental disorders.

Following the modernisation at the university hospital Würzburg we could drastically reduce the duration of stays in the intensive care unit to a mean of 1.5 days across around 500 admissions per year. Consequently, the risk of hospitalism especially for patients with chronic suicidality is practically non-existent anymore. Since 2017, our cooperation with other clinics in the region has made it possible to care for all underage patients requiring treatment in a protective intensive care unit in child and adolescent psychiatric settings. Long-term treatment over many months in a protective intensive care unit no longer occurs in cases of chronic suicidality.

## Einleitung

Der Suizid ist – nach Unfällen – die zweithäufigste Todesursache Minderjähriger in Deutschland. In bis zu 50 % der Fälle, in denen ein Suizidversuch zu einer klinischen Behandlung führt, kommt es im Verlauf zu einem erneuten Suizidversuch [1]. Das Risiko für ein Rezidiv ist innerhalb der ersten 6 Monate nach dem ersten Suizidversuch am höchsten. Von den Jugendlichen, die selbstverletzendes Verhalten zeigen, suizidieren sich im weiteren Lebensverlauf bis zu 4 % [2, 3]. Ihr Risiko, einen Suizid zu begehen, ist somit mehr als 100fach höher als in der Normalbevölkerung. Die Gefahren für die Betroffenen, die sich nicht nur aus dem suizidalen Ereignis selbst ergeben, sondern aus den Gesundheitsgefährdungen in den Wochen und Monaten danach, verdeutlichen die Bedeutung einer raschen Krisenintervention einschließlich der Einleitung therapeutischer sowie psychosozialer Maßnahmen.

2016 wurden die überarbeiteten Leitlinien der Deutschen Gesellschaft für Kinder- und Jugendpsychiatrie, Psychosomatik und Psychotherapie (DGKJP) zu Suizidalität sowie zu nichtsuizidalem selbstverletzenden Verhalten (NSSV) im Kindes- und Jugendalter veröffentlicht [4]. In der aktuell in Erstellung befindlichen Leitlinie der Arbeitsgemeinschaft der Wissenschaftlichen Medizinischen Fachgesellschaften e. V. (AWMF) zur Vermeidung von Zwang werden darüber hinaus Strategien zur Reduktion von Freiheitsentzug dargestellt. Die Leitlinien stellen auf Basis des aktuellen wissenschaftlichen Standes praxisorientierte Handlungsempfehlungen für die Behandlung dar.

Bei akuter Suizidalität und fehlender Absprachefähigkeit stellt die freiheitsentziehende Unterbringung (FEU) die Ultima Ratio im Sinne einer letztmöglichen Option zur Prävention von Suiziden dar. Da die FEU eine erhebliche Einschränkung der Grundrechte der Patienten darstellt, sind strenge rechtliche Rahmenbedingungen definiert. Bei der Unterbringung von Patienten mit psychischen Erkrankungen und Personen in akuten psychischen Ausnahmesituationen sind insbesondere bei minderjährigen Schutzbefohlenen hohe fachliche und ethische Normen anzuwenden. Die Eingangsvoraussetzung für die FEU besteht in einer akuten Eigen- oder Fremdgefährdung. Ziel dieser Maßnahme muss immer die Abwehr von Schaden sein, einmal bezogen auf die Person in der Krisensituation selbst oder – deutlich seltener – bei akuter Fremdgefährdung die unmittelbare Abwehr der Schädigung Dritter.

Eine akute Eigengefährdung meint im psychiatrischen Kontext in der Regel eine unmittelbare suizidale Krise, welche höchst unterschiedliche ätiologische Hintergründe und ebenso unterschiedliche zeitliche Dynamiken aufweisen kann. Sie kann im Verlauf psychischer Störungen auftreten, wie etwa bei Depression oder emotional instabiler Persönlichkeitsstörung, oder auch ohne vorbestehende psychische Störung, wie z. B. im Rahmen eines persönlichen Verlustes oder in Beziehungskrisen. Entsprechend können sich suizidale Krisen über die Zeit aufbauen oder plötzlich hoch akut entstehen.

Vor einer FEU sind zunächst sämtliche mildere Maßnahmen zum Schutz der Betroffenen auszuschöpfen. Nach zivilrechtlicher Definition sind seit der Novellierung des § 1631b BGB vom 01.10.2017 auch sogenannte „freiheitsentziehende Maßnahmen“ (FEM) als „milder“ einzustufen, auch wenn eine körperliche Fixierung, die den FEM zugerechnet wird, subjektiv als einschneidender erlebt werden kann als eine FEU. Seit der Novellierung sind FEM damit auch im geschlossenen, offenen oder auch ambulanten Setting unabhängig von einer FEU richterlich genehmigungspflichtig. Diese externe Kontrolle von FEM wurde von den kinder- und jugendpsychiatrischen Verbänden lange gefordert. Für die klinische Praxis haben sich daraus zum Teil erhebliche Veränderungen ergeben.

In dem vorliegenden Artikel werden fachliche, organisatorische und strukturelle Veränderungen der stationären Akutversorgung der letzten Jahre beispielhaft anhand der Neuorganisation einer geschlossen-stationären kinder- und jugendpsychiatrischen Versorgungsstation dargestellt. Diese Veränderungen sind wesentliche Voraussetzung für anstehende Aufgaben der Sicherstellung der Versorgung in regionalen Netzwerken mit distribuierten Verantwortlichkeiten.

## Entwicklung der rechtlichen Grundlagen von Freiheitsentzug

Seit der Einführung des § 1631b BGB im Jahr 1980 ist eine freiheitsentziehende Unterbringung eines Kindes oder Jugendlichen auf Antrag der Sorgeberechtigten nur mit richterlicher Genehmigung möglich. Dies wird u. a. damit begründet, dass eine FEU derart in die Grundrechte des Kindes einschneidet, dass die alleinige Entscheidung der Eltern dafür nicht ausreichen kann. Daneben bestehen weitere rechtliche Grundlagen für Zwangsmaßnahmen, zum einen landesrechtlich im Sinne einer öffentlich-rechtlichen Anordnung gemäß den jeweiligen landesspezifischen Gesetzen, zum anderen zivilrechtlich im § 1800 BGB und § 1915 BGB und ergänzend auch bei einem rechtfertigenden Notstand gemäß § 34 StGB.

Kinder- und jugendpsychiatrische geschlossene Abteilungen gelten gemäß dem durch den Europarat eingesetzten „European Committee for the Prevention of Torture and Inhuman or Degrading Treatment or Punishment“ (CPT) als sogenannte Haftorte. Sie stellen in dieser Hinsicht Bereiche mit hoher Gefahr für Menschenrechtsverletzungen dar und sind daher hinsichtlich ihrer Verfahrensweisen besonders zu kontrollieren. Dies geschieht u. a. dadurch, dass auch geschlossene Abteilungen der Kinder- und Jugendpsychiatrie nicht nur von Besuchskommissionen (z. B. in Bayern gemäß Art. 37 BayPsychKHG (Bayerisches Psychisch-Kranken-Hilfe-Gesetz)) zur Überprüfung der Rechtmäßigkeit einer öffentlich-rechtlichen Unterbringung unangekündigt aufgesucht werden, sondern in gleicher Weise auch durch die Mitglieder des CPT inspiziert werden dürfen, um die Standards im Umgang mit Freiheitsentzug zu prüfen und die Gefahr für Missachtungen der Rechte von Patienten zu minimieren.

Während für Erwachsene im § 1906 Abs. 3 BGB eigene Vorschriften für eine „Zwangsbehandlung“ in Abgrenzung zur Unterbringung definiert wurden, blieb diese Thematik für Minderjährige lange unscharf geregelt. Erst das Urteil des Bundesgerichtshofes vom 07.08.2013, dass Erziehungsberechtigte eigenständig Maßnahmen mit „freiheitsentziehendem Charakter“ im offenen Setting genehmigen konnten, diese also keiner richterlichen Genehmigungspflicht unterlagen [5], verdeutlichte die fehlende externe Kontrolle bei „unterbringungsähnlichen Maßnahmen“ bei Kindern und Jugendlichen (wie z. B. Festhalten, Einschluss im Zimmer oder auch Fixierungen) und in anderen Situationen mit „freiheitsentziehendem Charakter“ (z. B. medikamentöse Sedierung). Die Verbände BKJPP (Berufsverband für Kinder- und Jugendpsychiatrie, Psychosomatik und Psychotherapie in Deutschland e. V.), BAG KJPP (Bundesarbeitsgemeinschaft der Leitenden Klinikärzte für Kinder- und Jugendpsychiatrie, Psychosomatik und Psychotherapie e. V.) und DGKJP (Deutsche Gesellschaft für Kinder- und Jugendpsychiatrie, Psychosomatik und Psychotherapie e. V.) forderten daraufhin eine rechtliche Definition von FEM bei Kindern und Jugendlichen mit psychischen Erkrankungen, welche schließlich mit der Novellierung des § 1631b BGB im Jahr 2017 realisiert wurde. Durch die klare rechtliche Trennung zwischen der „Freiheitsentziehenden Unterbringung“ (Abs. 1) und „Freiheitsentziehenden Maßnahmen“ (Abs. 2) gilt nunmehr der Richtervorbehalt bei Minderjährigen für alle Arten von FEM wie Fixierung, Isolierung, Festhalten und sedierende Zwangsmedikation (siehe auch Definition der DGPPN (Deutsche Gesellschaft für Psychiatrie und Psychotherapie, Psychosomatik und Nervenheilkunde e. V.) [6]).

## Auswirkungen der Novellierung des § 1631b BGB auf die Praxis

Die dargestellte Neuregelung hat zu weitreichenden Veränderungen in der bisherigen Praxis geführt, da nunmehr die Anwendung oder das Vorhalten von FEM (§ 1631b BGB, Abs. 2) von der Notwendigkeit einer FEU (§ 1631b BGB, Abs. 1) entkoppelt wurde. Beide Maßnahmen müssen voneinander unabhängig geprüft werden. Das bedeutet beispielsweise, dass die Notwendigkeit zur regelmäßigen Fixierung bei einem Kind, bei dem aufgrund von akuter Suizidalität eine FEU durch die sorgeberechtigten Eltern beim Familiengericht erwirkt wurde, einen weiteren Beschluss nach Abs. 2 für FEM erforderlich macht. Wichtig ist, dass die Notwendigkeit von regelmäßigen Fixierungen oder Time-out-Maßnahmen (kurzzeitige Isolierung in einem reizarmen Raum) im Gegenzug keine ausreichende Begründung für eine FEU per se darstellt. Zudem unterstreicht das Bundesverfassungsgericht, dass bei der Anwendung von FEM grundsätzlich immer die am wenigsten eingreifende Maßnahme gewählt werden muss [7]. Auch wird jede FEM unzulässig, sobald sie zum Wohl des Kindes nicht mehr notwendig ist oder der Gefahr für das Kind auf eine andere (mildere) Weise begegnet werden kann (siehe Gesetzesbegründung, [8]). Dies führt in der Praxis dazu, dass täglich fachärztlich überprüft werden muss, ob die Erfordernis für FEM und/oder FEU noch besteht.

Auch wenn die gesetzlichen Regelungen nun erfreulicherweise in vielen Punkten zu Klarheit und Rechtssicherheit geführt haben, bleiben doch viele Details offen. Da FEM vor der Novellierung nicht flächendeckend statistisch erhoben wurden, kann letztlich bis heute nicht sicher nachgewiesen werden, ob nach der Gesetzesänderung eine generelle Reduktion der Anwendung von FEM zu verzeichnen ist. Zumindest in Bezug auf FEM in bayerischen Einrichtungen für Kinder und Jugendliche mit Intelligenzminderung konnte dies in eigenen Erhebungen nicht belegt werden [9]. Auch bleibt z. T. die Frage offen, wo die Grenze zwischen pädagogischer Maßnahme in Verantwortung der Sorgeberechtigten und einer FEM mit richterlichem Genehmigungsvorbehalt verläuft. Wann dient eine Zwangsmedikation dem Zweck, das Kind am Verlassen eines Ortes zu hindern, wann ist es eine therapeutische Maßnahme mit Sedierung als Nebenwirkung? Auch die Einschätzung, ab wann von einer im Gesetz formulierten „Regelmäßigkeit“ der Anwendung von FEM auszugehen ist, wird durch den Gesetzestext nicht klar definiert und ist letztlich von der Einschätzung des jeweiligen Familienrichters abhängig [7]. Für die Kliniker folgt aus den bestehenden Unklarheiten, dass hinsichtlich dieser Aspekte möglichst klare Handlungsvorgaben zu definieren sind.

## Umgestaltung der Intensiveinheit am Universitätsklinikum Würzburg

Für die Erfüllung des Sicherstellungsauftrages in der Versorgung von Kindern und Jugendlichen mit akutstationärem psychiatrischen Behandlungsbedarf im Bezirk Unterfranken wurde 2004 in Würzburg die „Intensiv-Therapie-Einheit“ (ITE) eröffnet (Trägerschaft: 2004 bis 2022 Bezirk Unterfranken, seit 2023 Universitätsklinikum Würzburg). Ursprünglich wurden 6 Plätze für die Akutversorgung der ca. 200.000 dort lebenden Minderjährigen vorgehalten.

Jedoch war diese Kapazität angesichts der stetig steigenden Inanspruchnahme nicht ausreichend, um den Sicherstellungsauftrag vollumfänglich zu erfüllen, sodass weiterhin in den regionalen erwachsenenpsychiatrischen Bezirkskrankenhäusern Jugendliche untergebracht werden mussten. Dies führte dazu, dass z. B. im Jahr 2014 fast 200 Minderjährige vorübergehend auf den geschlossenen Stationen in Lohr und Werneck aufgenommen wurden, was aufgrund der unterschiedlichen rechtlichen und fachlichen Anforderungen in den Kliniken zu hohem Aufwand führte und für alle Seiten nicht zufriedenstellend war. In der Folge wurde die Bettenzahl der Intensiveinheit in Würzburg sukzessive von 6 auf 14 erweitert. Erst 2023 konnte durch Schaffung fakultativ-geschlossener Plätze in den kinder- und jugendpsychiatrischen Fachkliniken in Schweinfurt und Aschaffenburg dem hohen Bedarf an Akutversorgung Rechnung getragen und eine Regionalisierung der stationären Akutversorgung in Unterfranken vorangetrieben werden.

Mit dem Ausbau der stationären Kapazitäten seit 2004 erfolgte in den letzten Jahren eine fachliche und strukturelle Neukonzeption der Akutversorgung. Diese verfolgte u. a. folgende Ziele:

- Minimierung von Freiheitsentzug und Zwang in der Akutbehandlung,
- Sicherstellung der akutstationären kinder- und jugendpsychiatrischen Versorgung in Unterfranken ohne Inanspruchnahme erwachsenenpsychiatrischer Klinikstrukturen,
- Verbesserung der Partizipation der Patienten,
- Reduktion von Hospitalisierungen bei chronischer Suizidalität,
- Verbesserung der Verzahnung mit Strukturen der Jugendhilfe.

Bei der Umstrukturierung mussten verschiedene fachliche, organisatorische und baulich-strukturelle Maßnahmen ergriffen und aufeinander abgestimmt werden.

### Kinderschutzkonzept.

Durch eine interdisziplinäre Arbeitsgruppe der Klinik wurde ein Schutzkonzept erarbeitet, welches die UN-Kinderechtskonvention als international bindende rechtliche Norm zur Grundlage hat. Das Konzept wurde im Jahr 2017 verpflichtend für alle klinischen Mitarbeiter implementiert. Im Kontext der FEU und der Anwendung von FEM bei minderjährigen Patienten sind folgende Artikel von besonderer Bedeutung: „Achtung der Kinderrechte“ (Art. 2), „Wohl des Kindes“ (Art. 3), „Berücksichtigung des Kindeswillens“ (Art. 12), „Schutz der Privatsphäre und der Ehre“ (Art. 16), „Schutz vor Gewaltanwendung“ (Art. 19) und die „regelmäßige Überprüfung der Umstände der Unterbringung“ (Art. 25).

Das Kinderschutzkonzept ist in einer Broschüre auf der Homepage der Klinik öffentlich zugänglich [10]. Zentral sind u. a. die Analyse von Gefährdungsmomenten, z. B. ein transparenter Verhaltenskodex sowie Interventionsstandards zum Umgang mit möglichen Tätern und Opfern. In Kinderschutzfragen kann z. B. von jedem Mitarbeiter ein multiprofessionelles Kinderschutz-Krisenteam u. a. bestehend aus den therapeutischen Mitarbeitern sowie Pflegekräften einberufen werden, das den eingebrachten Fall zeitnah bespricht. Auch erfolgt am Ende des stationären Aufenthaltes mit dem „Würzburger Fragebogen zur Qualitätssicherung in der stationären Versorgung“ eine Abschlussbefragung der Patienten zur Behandlungszufriedenheit, in der auch mögliche emotionale, körperliche und sexuelle Missbrauchserfahrungen direkt thematisiert werden. So soll eine „Kultur des Hinschauens“ in der Klinik weiter gefestigt werden. Zusätzlich wurde dafür in einer retrospektiven Nachbefragung ehemaliger Patienten zu Erfahrungen emotionaler, körperlicher und sexueller Gewalt in der stationären Behandlung ein weiterer Grundstein gelegt [11].

### Fachliche Qualifizierung des Teams.

Die Klinikmitarbeiter wurden seit dem Jahr 2014 kontinuierlich in der „Dialektisch Behavioralen Therapie für Adoleszente“ (DBT-A) fortgebildet und qualifiziert. Dies führte zu einer Veränderung der Haltung im Team hin zu einer stärkeren Klientenzentrierung und Fokussierung auf das krisenhafte Verhalten. Grundsätzlich wird dazu zunächst die suizidale Krise zusammen mit dem Patienten analysiert (Verhaltens‑/Kettenanalysen) und es werden Frühwarnzeichen für akute vs. chronische Suizidalität erarbeitet. Anschließend werden u. a. gemeinsam Notfallpläne entwickelt, um Suizidgedanken und -versuchen vorzubeugen. Schließlich wird am Commitment für eine ambulante Behandlung gearbeitet. Durch dieses Vorgehen wird eine transsektorale Überführung in ein stabiles ambulantes Behandlungssetting ermöglicht. Zudem werden die Mitarbeiter aller Klinikbereiche kontinuierlich im Einsatz von Deeskalationsstrategien mittels der ProDeMa-Methodik (Professionelles Deeskalationsmanagement; [12]) geschult.

### Zusammenarbeit mit Familienrichtern.

In Antizipation der Novellierung des § 1631b BGB wurden im Jahr 2017 verschiedene Treffen zwischen den zuständigen Familienrichtern und der Leitung der Klinik organisiert. Es wurden zahlreiche Fallbeispiele hinsichtlich der Notwendigkeit des Einholens einer richterlichen Genehmigung intensiv diskutiert und noch vor Inkrafttreten der Novellierung einheitliche Standards festgelegt, obgleich die richterliche Freiheit jederzeit abweichende Entscheidungen ermöglicht.

### Anpassung der Indikationen für Freiheitsentzug.

Vor dem Hintergrund der Frage nach der Verhältnismäßigkeit von FEU und der erforderlichen Prüfung, ob mildere Maßnahmen hinreichend wären, wurde auch die Indikationsstellung für eine Aufnahme auf die ITE grundlegend überarbeitet. So wurden die Eingangskriterien für eine FEU rechtskonform definiert, um sicherzustellen, dass die Kriterien für FEU und FEM grundsätzlich kritisch und separat voneinander geprüft werden. Die Notwendigkeit z. B. von Fixierungen oder Zwangsernährung von Patienten allein war damit nicht hinreichend für eine FEU, da diese grundsätzlich auch im offen-stationären Setting durchführbar sind. Auch nichtsuizidales selbstverletzendes Verhalten ohne bestehende akute Suizidalität stellt keine hinreichende Begründung für FEU dar. Diese Entwicklung wurde begleitet durch die Einrichtung und den sukzessiven Ausbau einer „AtRisk“-Spezialambulanz für Jugendliche mit Risikoverhalten (sprich: „at risc“), insbesondere bei Suizidalität und NSSV. Neben einer differenzierten Diagnostik wird in der Spezialambulanz auch eine Kurzzeit-Psychotherapie nach DBT‑A angeboten. Insbesondere die tägliche Überprüfung der Notwendigkeit der Fortführung einer FEU (in Abgrenzung von FEM) führte in diesem Versorgungsnetzwerk zu einer deutlichen Reduzierung der Liegezeiten auf der ITE.

Die Reduktion von Freiheitsentzug und die damit einhergehende Verkürzung der durchschnittlichen Liegezeiten offenbarte eine Versorgungslücke für Minderjährige, die nach Überwinden der akuten Suizidalität einen weiteren stationären Behandlungsbedarf haben, da die offen-stationären Kapazitäten zunächst limitiert waren. Nach Entaktualisierung der Krisensituation mit akuter Suizidalität muss angesichts der strengen rechtlichen Rahmenbedingungen eine unmittelbare Entlassung der Patienten aus dem geschlossenen Setting erfolgen. Eine Weiterführung der Behandlung ist im Anschluss nur bei bestehender Freiwilligkeit möglich, da nach Rechtsauffassung der Würzburger Familienrichter eine freiwillige Behandlung im geschlossenen Setting unzulässig ist. Dieser Rechtsauffassung schließen wir uns vollumfänglich an, zumal eine „freiwillige“ geschlossene Behandlung Machtmissbrauch befördert und eine faktische Umgehung von Rechtsvorschriften ermöglicht („Bleibst Du freiwillig oder müssen wir den Richter holen?“). Ein Verbleib im geschlossenen Setting nach Überwinden der unmittelbaren und akuten Suizidalität birgt zudem die Gefahr von Hospitalisierungen und verlängerte stationäre Verläufe mit der Gefahr einer iatrogenen Schädigung der Patienten. Aus rechtlicher sowie aus fachlicher Sicht muss daher eine klare und unzweideutige Trennung dahingehend erfolgen, ob und wann die Voraussetzungen für die Anwendung des § 1631b Abs. 1 BGB vorliegen und wann nicht (mehr). Der beschriebene Versorgungsengpass konnte durch strukturelle und organisatorische Anpassungen überwunden werden.

### Strukturelle und organisatorische Anpassungen.

Vor dem Hintergrund der genannten Versorgungslücke erfolgte ein struktureller Umbau der ITE verbunden mit einer organisatorischen Neukonzeption, wodurch eine über die Krisenbewältigung hinausgehende kinder- und jugendpsychiatrische Weiterbehandlung von Intensivpatienten ermöglicht wurde. Es wurden dazu innerhalb der ITE 2 separate Funktionsbereiche geschaffen, die in ihrer Größe flexibel und an den situativen Bedarf anpassbar in einen „Schutzbereich“ sowie einen „Therapiebereich“ unterteilt werden können.

**Figure 1:**
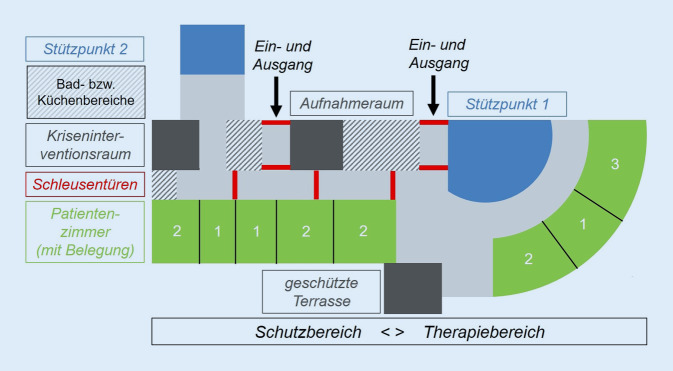

Dies wurde technisch durch den Einbau zusätzlicher Schleusentüren möglich, wodurch abhängig vom Bedarf die Größe der entsprechenden Bereiche variiert werden kann (Abb. 1). Der Schutzbereich dient der Aufnahme von Patienten in akut-psychiatrischen Notfallsituationen mit Indikation für eine FEU nach § 1631b Abs. 1 BGB. Hauptmerkmal dieses Bereiches ist der Schutz der akut suizidalen bzw. deutlich herabgesetzt steuerungsfähigen Patienten mit Eigen- und/oder Fremdgefährdung. Er dient neben der initialen Krisenintervention auch einer ersten diagnostischen Einschätzung mit Erstellung von weitergehenden Behandlungsempfehlungen und Prüfung des Behandlungscommitments. Die funktionelle Abtrennung des geschlossenen Schutzbereiches von einem offenen Therapiebereich ermöglicht eine stationäre Weiterbehandlung der weiterhin zumeist hoch instabilen Akutpatienten mit intensivem Therapiebedarf auch jenseits der Krisenintervention. Wesentlicher Vorteil dieser Konzeption ist, dass weder die Station noch das Behandlungsteam wechselt und die Behandlungskontinuität von der akuten Krise bis zur Entlassung aus dem stationären Setting gewährleistet werden kann.

**Figure 2:**
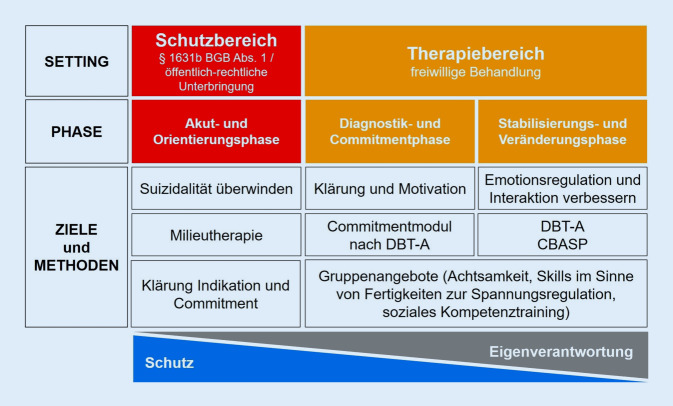

Durch die interne Strukturierung ergeben sich im Diagnostik- und Therapieprozess unterschiedliche Phasen mit unterschiedlichen Schwerpunkten (Abb. 2).

### Kooperationsvereinbarungen mit der Jugendhilfe.

Etwa 20 % der stationär behandelten Kinder und Jugendlichen in Bayern leben in Einrichtungen der Jugendhilfe. Bei weiteren 20 % wird im Rahmen der teil-/stationären Behandlung fachärztlich die Indikation und Empfehlung einer stationären Jugendhilfemaßnahme festgestellt [13]. Diese Zahlen belegen die hohe Schnittmenge im Klientel der Jugendhilfe und KJPPP, welche charakterisiert ist durch das parallele Bestehen von kinder- und jugendpsychiatrischem Behandlungsbedarf, (sonder-/sozial-)pädagogischem Erziehungsbedarf und hohem Wiedereingliederungsbedarf in schulische bzw. familiäre und außerfamiliäre soziale Strukturen. Die komplexen Bedarfe der Kinder und Jugendlichen machen eine enge Verzahnung der Strukturen erforderlich, weshalb in vielen Regionen enge Kooperationen zwischen Kinder- und Jugendpsychiatrie und Kinder- und Jugendhilfestrukturen entstanden sind. Auch im „Würzburger Modell“ sind diese bereits vor mehr als 25 Jahren u. a. durch ein ausgedehntes Konsiliardienstsystem in stationären Einrichtungen der Jugend- sowie Behindertenhilfe realisiert worden. Die Kooperation ermöglicht die Vermeidung stationärer Aufnahmen und ambulanter Krisenintervention in der Klinik, reduziert stationäre Liegezeiten und befähigt die Jugendhilfe, auch Kindern und Jugendlichen mit hochkomplexen Fallkonstellationen in intensiv-therapeutischen Wohngruppen einen angemessenen Rahmen bereitzustellen. In der gemeinsamen Verantwortungsübernahme gilt es insbesondere an der Grenze der Systeme einerseits Krisensituationen in der stationären Jugendhilfe strukturiert und fachlich in der Klinik zu begegnen und andererseits eine rasche Rückführung bzw. erstmalige Überführung von Patienten in bedarfsgerechte Jugendhilfemaßnahmen zu gewährleisten. Hierbei müssen die fachlichen sowie rechtlichen Grundsätze zu jedem Zeitpunkt gewahrt bleiben. So rechtfertigt die fehlende Bereitschaft eines Minderjährigen, in einer offenen Jugendhilfeeinrichtung zu verbleiben, nicht per se eine FEU in einer kinder- und jugendpsychiatrischen Klinik, sodass nach Ausschluss von akuter Eigen- oder Fremdgefährdung an dieser Stelle auf entsprechende geschlossene Einrichtungen der Jugendhilfe verwiesen werden muss.

### Stationäre Akutversorgung im gestuften Versorgungsnetzwerk.

Inhaltlich empfehlen die genannten Leitlinien im Kontext psychiatrischer Akutsituationen ein graduell gestuftes Vorgehen (*Stepped-care*), d. h., dass abhängig von der aktuellen Symptomatik die jeweils angemessene Intervention im jeweils angemessenen Setting angeboten wird. Diese Logik dient einerseits dazu, geeignete und ökonomisch sinnvolle Hilfestellungen zu leisten und andererseits stark einschneidende Maßnahmen wie FEU und FEM nur bei strenger Indikationsstellung anzuwenden.

Entsprechend wurde innerhalb der Intensiveinheit ein leitlinienkonformes gestuftes Versorgungsmodell für Patienten mit akutem Behandlungsbedarf und hoher Rückfallgefahr realisiert. Die stationäre Akutversorgung in Deutschland ist jedoch eingebettet in regional höchst unterschiedlich ausgestaltete Versorgungssituationen, die mehr oder weniger stark untereinander kooperativ verschränkt und letztlich für ihr jeweiliges Funktionieren voneinander abhängig sind. Für die Reduktion stationärer Behandlungsphasen von Patienten mit NSSV in der Würzburger Klinik war der parallele Aufbau der AtRisk-Spezialambulanz (siehe oben) unabdingbar. Diese ist eingebettet in die Bayern-spezifische Versorgungsstruktur der psychiatrischen Institutsambulanzen, deren Behandlungsauftrag u. a. die Versorgung von Patienten mit chronischer und/oder komplexer psychischer Symptomatik ist. Übergeordnetes Ziel ist, stationäre Krankenhausaufnahmen zu vermeiden. Eine solche klinische Versorgungsstruktur hat im Übrigen nur bei einer ausreichenden Zahl von niedergelassenen kinder- und jugendpsychiatrischen/-psychotherapeutischen Kollegen auch ausreichend Kapazitäten für Spezialisierungen für derart betroffene Kinder und Jugendliche. Auch sind detaillierte Absprachen und Kooperationsmodelle erforderlich, um die gemeinsame Verantwortung für Kinder und Jugendliche mit komplexen Bedarfen strukturell abbilden zu können und zu gewährleisten.

**Figure 3:**
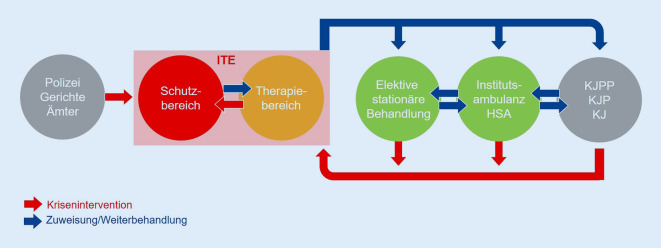

Die hier beschriebene strukturelle Modifikation kann als exemplarisches Modell für eine solche Vernetzung regionaler Versorgungstrukturen angesehen werden, wobei bereits die klinischen Strukturen im Kontext von Kriseninterventionen und deren Nachsorge eng verzahnt sein sollten (Abb. 3).

Zusammenfassend wird durch das Vorhalten einer Versorgungskette von geschlossener Krisenintervention über eine intensivtherapeutische Behandlungsoption, teil-/stationärer Regelbehandlung hin zu ambulanter Therapie ein innovatives gestuftes Konzept ermöglicht. Bei einer hohen Überlappung zwischen Kindern und Jugendlichen in vor allem stationären Jugendhilfemaßnahmen und den Patienten der ITE sind eine enge kooperative Verzahnung und Abstimmung komplementärer Versorgungsstrukturen geschaffen worden.

## Fazit

Die Novellierung des § 1631b BGB hatte katalytische Wirkung auf die Modernisierung der dargestellten Versorgungsstrukturen. Freiheitsentzug konnte auf ein Minimum reduziert werden, auf aktuell durchschnittlich 1,5 Tage bei etwa 500 Aufnahmen mit FEU im Jahr. Die detaillierte Statistik zu dieser Entwicklung wird an anderer Stelle publiziert werden (Geißler et al. in Vorbereitung). Durch die Neukonzeption konnte auch die erhebliche Gefahr der Hospitalisierung insbesondere bei chronisch suizidalen Patienten deutlich verringert werden. Das Ziel, alle Patienten mit akutstationärem Behandlungsbedarf in kinder- und jugendpsychiatrischen Strukturen zu versorgen, konnte in Kooperation mit den Fachkliniken in Schweinfurt und Aschaffenburg erreicht werden. Seit 2017 wurde kein Minderjähriger mehr in den erwachsenenpsychiatrischen Versorgungskliniken der Region aufgenommen. Verläufe mit geschlossen-stationärer Behandlung über viele Monate oder gar Jahre bei chronischer Suizidalität kommen in Würzburg nicht mehr vor.
